# Developments in Vaccination for Herpes Simplex Virus

**DOI:** 10.3389/fmicb.2021.798927

**Published:** 2021-12-07

**Authors:** Rohini Krishnan, Patrick M. Stuart

**Affiliations:** Department of Ophthalmology, Saint Louis University School of Medicine, St. Louis, MO, United States

**Keywords:** vaccines, herpes simplex virus type 1, herpes simplex virus type 2, mRNA vaccine, live attenuated vaccine, subunit vaccine, DNA vaccine

## Abstract

Herpes simplex virus (HSV) is an alpha herpes virus, with two subtypes: HSV-1 and HSV-2. HSV is one of the most prevalent sexually transmitted infections. It is the cause of severe neonatal infections and a leading cause of infectious blindness in the Western world. As of 2016, 13.2% of the global population ages 15–49 were existing with HSV-2 infection and 66.6% with HSV-1. This high prevalence of disease and the fact that resistance to current therapies is on the rise makes it imperative to develop and discover new methods of HSV prevention and management. Among the arsenal of therapies/treatments for this virus has been the development of a prophylactic or therapeutic vaccine to prevent the complications of HSV reactivation. Our current understanding of the immune responses involved in latency and reactivation provides a unique challenge to the development of vaccines. There are no approved vaccines currently available for either prophylaxis or therapy. However, there are various promising candidates in the pre-clinical and clinical phases of study. Vaccines are being developed with two broad focuses: preventative and therapeutic, some with a dual use as both immunotherapeutic and prophylactic. Within this article, we will review the current guidelines for the treatment of herpes simplex infections, our understanding of the immunological pathways involved, and novel vaccine candidates in development.

## Introduction

Herpes simplex virus (HSV) is a prevalent sexually transmitted infection, a leading cause of infectious blindness in the Western world, and is the most common cause of focal, sporadic encephalitis in the United States. It is also a significant cause of neonatal mortality (Whitley and Green, 2019).

As of 2016, 13.2% of the global population aged 15–49 were existing with HSV-2 infection and 66.6% with HSV-1 (James et al., 2020). This high prevalence of disease and the fact that resistance to current therapies is on the rise makes it imperative to develop and discover new methods of HSV prevention and management. Vaccination has been shown to effectively reduce the spread of viral infections and increasing herd immunity (Johnston et al., 2016).

Currently, there are no vaccines approved for prevention of HSV infection after years of development and innovation. Herpesviruses are known to establish latency, which complicates vaccine development as an effective vaccine must not only prevent active clinical disease, but ideally latent infection as well. Development of the therapeutic Shingrix vaccine for Herpes Zoster and a prophylactic vaccine for varicella zoster virus (VZV) has increased efforts for HSV vaccine due to similarities between the two viruses, especially with the establishment of latency (Heineman et al., 2019). However, key differences in the immune responses elicited by VZV and HSV and immune evasion mechanisms complicate the advances toward an effective vaccine for HSV.

## Herpes Simplex Virus

**Figure 1:**
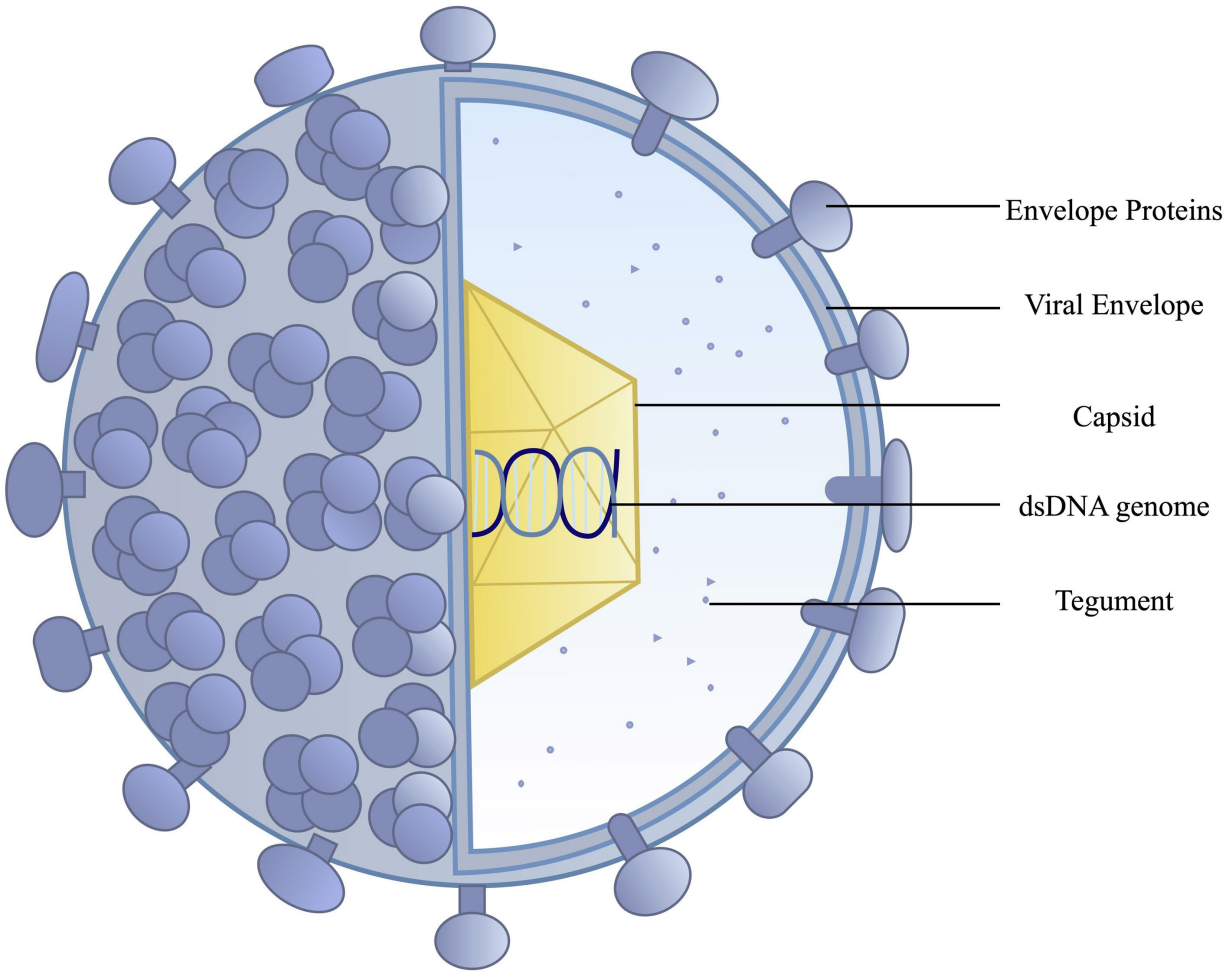
Herpes simplex viruses have an outer lipid bilayer envelope with an inner icosahedral capsid. Mature virions contain an amorphous layer of proteins, the tegument, outside the nucleocapsid. The lipid bilayer envelope, derived from the host cell membrane, contains a range of viral glycoproteins.

HSV is an alpha herpes virus with a dsDNA genome with two main subtypes, HSV-1 and HSV-2. The main components surrounding the dsDNA core include an icosapentahedral capsid (100–110 nm) anchored by 20–23 tegument proteins. These tegument proteins provide structure and are beneficial to viral proliferation during infection, contributing to immune evasion (Maruzuru et al., 2018). These tegument proteins serve as an additional target for vaccine development, as immune recognition of these proteins could limit viral pathogenesis. The viral capsid is surrounded by a lipid bilayer of polyamines and around 12 glycoproteins (gB, gC, gD, gE, gG, gH, gI, gJ, gK, gL, gM, and gN; Madavaraju et al., 2021). These viral glycoproteins are common vaccine targets as they are essential to viral fusion with the cell membrane and can contribute to immune system evasion. Glycoprotein D is a common target for most vaccines as it is essential for viral entry into host cells (Whitbeck et al., 1997). Figure 1 depicts the structure of herpes simplex virus.

**Figure 2:**
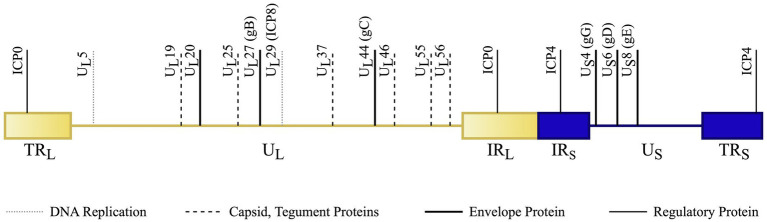
HSV genome schematic (not to scale) with notable vaccine targets. The dsDNA genome is divided into the U_L_ (unique long sequence – light, shaded yellow) and U_S_ (unique short – dark blue). Short regions of repeated sections occur at the ends of each of these sequences. Terminal repeats (TR_L_ and TR_S_) occur at the ends of the genome and internal repeats (IR_L_ and IR_S_) flank the transition between the long and short segments. Vaccines are developed with broad targets involving envelope, capsid, tegument, DNA replication, and regulatory proteins. The genes coding for these protein targets are specifically denoted in the above image. Original Image. References: (Dolan et al., 1998; Lim et al., 2013; Jiao et al., 2019; Denes et al., 2020).

HSV-1 and 2 have approximately 50% genomic homology (Berger and Houff, 2008); therefore, most vaccine targets for HSV-1 or 2 can often provide cross-protection for both subtypes. Notably, antigen specificity is derived from glycoprotein G (gG), which helps distinguish the two types of HSV (Whitley and Green, 2019). Although HSV-1 has predominantly been linked to ocular infection and HSV-2 with genital infection, that characterization is changing with the knowledge that an increasing proportion of some populations (above 50%) have HSV-1 derived genital infections (Whitley and Green, 2019). Figure 2 depicts the various genes and proteins in the HSV genome that have been modified, deleted or targeted during vaccine development.

### HSV Pathogenesis and Replication

Replication is a multi-step process. Post-infection, HSV glycoproteins interact with and attach to the cell. The viral envelope then fuses with the cell membrane, releasing its contents into the cell. DNA is uncoated and transported into the nucleus when nucleocapsid fuses with the viral envelope (Madavaraju et al., 2021). Here, immediate-early genes are transcribed (regulatory proteins, enzymes). Primary herpes infection involves replication within epithelial cells, with an incubation period of 4–6 days. Mature virions are then transported to the cell membrane, where they are released, causing cell lysis and local inflammation. Replication continues until host immune responses contain the initial infection (Whitley and Green, 2019).

Herpesvirus ascends peripheral sensory nerves to spread to the trigeminal ganglion, where latent infection develops. Latent virus can be induced to reactivate. Reactivation has been associated with various stimuli including stress, infections, and UV light exposure. However, from a molecular standpoint, reactivation is a phenomenon that continues to be studied and is not clearly understood. Clinical manifestations of reactivation vary widely in terms of presentation and severity (Whitley and Green, 2019).

### Clinical Herpes Simplex Infection

Primary infection with herpes simplex involves grouped vesicles on an erythematous base. Mucocutaneus manifestations include eczema herpeticum, herpetic whitlow, herpes gladiatorum, folliculitis, or a severe/chronic mucocutaneous infection. Extracutaneous manifestations include ocular HSV keratoconjunctivitis. Herpes Encephalitis, Proctitis, and Neonatal HSV. These manifestations are often the ones with prominent morbidity and mortality (Bolognia et al., 2018).

Ocular HSV infection has complications that include corneal ulceration and scarring, globe rupture, and subsequent blindness. Epithelial keratitis has strong links to viral replication, while stromal keratitis is primarily immunopathologic (due to stromal antigen) and is promoted mainly by T cells (Russell et al., 1984). Consequently, HSV is one of the main causes of infectious blindness in developed countries (Valerio and Lin, 2019).

Herpes encephalitis has >70% mortality without treatment, with residual neurological defects in most surviving patients (Jouan et al., 2015). Similarly, disseminated neonatal HSV has 50% mortality without treatment, 15% with treatment and with neurologic deficits in many recovering patients (Bolognia et al., 2018).

### Primary and Secondary Immune Responses to HSV

#### Innate Immune Response

The innate immune system consists of a variety of components including neutrophils, natural killer cells, monocytes, dendritic cells, macrophages, and the complement cascade (Xu et al., 2019). It is the first line of defense against pathogens. A strong innate immune response to foreign antigens is essential to the development of the adaptive humoral and cellular responses of long-lasting immunity (Alberts et al., 2002). Adjuvants are added to subunit vaccines to elicit an innate response for this purpose. Pattern recognition receptors on host cells allow for the detection and response to viral infections. On binding to pattern recognition receptors (PRRs), signal transduction leads to the production of various proteins including interferons (Zhu and Zheng, 2020). Interferons are glycoprotein signaling molecules produced by infected cell lines. They act locally, priming viral defense mechanisms within local cells (downregulating protein synthesis, upregulating MHC expression).

HSV-1 is known to have multiple ligands that bind TLRs, leading to activation of the NF-kB pathway (Lund et al., 2003). However, herpes simplex virus is also known to evade the innate immune responses, specifically TLR signaling and interferon production to further viral proliferation (Villalba et al., 2012; Liu et al., 2013). Additionally, a critical aspect of the innate immune system that contributes to the adaptive response is antigen presentation. HSV infection can stimulate autophagy, leading to a more efficient presentation of antigens. However, HSV-1 ICP34.5 gene product antagonizes the response by binding Beclin 1 (Atg6), essential to autophagy (English et al., 2009; Leib et al., 2009).

#### Adaptive Immune Response

Unlike the innate immune system, the adaptive immune system is targeted to the pathogen and more sophisticated, conferring enduring protection. It is divided into humoral and cell-mediated responses that are carried out by either B-lymphocytes or T-lymphocytes, respectively. B cells are a vital arm of the humoral response which is the primary method for inactivated and subunit vaccines to confer protection in the form of neutralizing antibodies. These antibodies have a broad range of potential targets and have multiple protective actions by blocking viral receptors, marking pathogens for destruction *via* antibody-dependent cellular toxicity (Alberts et al., 2002). While HSV-specific IgG and mucosal IgA are seen with HSV infection (Hadar and Sarov, 1984), they are not effective at preventing HSV reactivation.

Cell-mediated responses have stronger supporting evidence for immunoprotection, as immunocompromised patients (with lower T-cell counts) have been shown to have more severe disease (Piret and Boivin, 2016). Cell-mediated responses are primarily elicited from live-attenuated vaccines and DNA/mRNA vaccines. T cells are activated by antigen-MHC complexes to produce specific effector responses that include CD4^+^ T-cell induced B-cell class-switching and CD8^+^ T-cell induced cytotoxicity. CD4^+^ Th1 cells are particularly notable for potentiating an effective immune response to viral pathogens (Alberts et al., 2002). The adaptive immune response has been implicated in various studies with the development of virus (HSV) specific CD4^+^ and CD8^+^ T cells (Zhu et al., 2007) during active infection and have been shown to persist for months after healing. Additionally, HSV latency in the human trigeminal ganglion has been associated with T-cell accumulation. Specifically, latent infection has been associated with CD8^+^ T-cell persistence in the ganglia likely from stimulation by parenchymal cells (Van Lint et al., 2005).

However, HSV-1 has been shown to have mechanisms impeding the CD8^+^ T-cell-mediated eradication of virus from latency (Verjans et al., 2007). Additionally, the density of CD8^+^ T cells in genital mucosa is predictive of the duration and severity of viral reactivation (Schiffer et al., 2010).

#### HSV Immune Evasion and Vaccine Development

As discussed earlier, HSV has multiple methods of immune evasion, involving both the innate and the adaptive immune system. Vaccine design has focused on structural proteins involved in immune evasion. A notable example includes glycoprotein C, which has been shown to regulate the complement cascade by binding C3, preventing cleavage of C3 to C3b, forming the membrane attack complex (MAC) and subsequent cell lysis (Hook et al., 2006).

### Approaches to Vaccine Development

Although there are no currently available vaccines for herpes simplex 1 and 2, there are various candidates in both the pre-clinical and the clinical phases currently in development. Vaccines are being developed with two broad focuses: preventative and therapeutic, some with a dual use. Preventative vaccines are focused on the prevention of primary infection in a seronegative subject. Therapeutic vaccines aim to prevent HSV reactivation, decrease the number of recurrences, or to reduce the severity or duration of clinical symptoms (Sela and Hilleman, 2004). With regard to vaccine development, given our knowledge of the immunology surrounding HSV, it seems that an effective vaccine would likely stimulate not only humoral responses, but also cell-mediated responses. Different vaccine subtypes (Live-attenuated, Recombinant, etc.) have their unique advantages and disadvantages, discussed further in the next section.

Recombinant vaccines are usually composed of proteins that are not strong immunoactivators. Therefore, they require adjuvants to stimulate the innate immune system. This leads to the humoral response and proper inoculation. They are not needed for live-attenuated viruses. Different constituents can enhance and target different facets of the immune response. It is important to focus on the adjuvants in each vaccine trial and evaluate their role in eliciting a lasting humoral and cell-mediated response (Truong et al., 2019).

## Vaccines in Development

Table 1 lists the vaccines in development in both the pre-clinical and the clinical arena.

**Table 1:** Vaccines in development for infection with HSV-1.

*Candidate name*	Developer	Vaccine Components and Methodology	Developmental Phase	References
*Live-Attenuated Vaccines*				
*HSV-2 ΔNLS (RVX 201)*	SIU - Rational Vaccines	Live, attenuated replication-competent HSV-2 with deletion of ICP0	Phase II - DC	Halford et al., 2010, 2011
*HSV-1 VC2*	Louisiana State University	HSV-1 with mutations in gK and UL20	Pre-Clinical	Stanfield et al., 2014, 2017, 2018
*R2*	Thyreos LLC	HSV-1 with pUL37 gene mutation at R2 region	Pre-Clinical	Richards et al., 2017; Bernstein et al., 2020
*HSV-2 ΔgD2*	Albert Einstein College of Medicine	HSV-2 with US6 (gD) deletion	Pre-Clinical	Petro et al., 2015, 2016
*AD472*		Deleted both copies of the γ134.5, UL55-56, UL43.5, and the US10-12 region	Pre-clinical	Prichard et al., 2005
*NE-HSV-2*	BlueWillow Biologics	Nanoemulsion with gB2 and gD2 antigens	Pre-Clinical	BlueWillow Biologics, 2019
*Subunit Vaccines*				
*HSV-2 trivalent vaccine*	University of Pennsylvania	gC2, gD2, gE2; CpG/alum Adjuvant	Clinical	Awasthi et al., 2011, 2017; Hook et al., 2019; Egan et al., 2020
*HSV1 gB lentiviral vector*	University of Pisa, Italy	Lentiviral vector expressing gB1	Pre-Clinical	Chiuppesi et al., 2012
*GEN-003*	Genocea	gD2 (truncated)+ICP4 fragment (29.2 kD)+Matrix M2 Adjuvant	Completed Phase II (DC)	Skoberne et al., 2013; Flechtner et al., 2016; Van Wagoner et al., 2018; Bernstein et al., 2019
*HerpV*	Agenus	32 synthetic 35mer HSV-2 peptides complexed with Hsc70 protein + QS21 (saponin adjuvant)	Phase II (DC)	Mo et al., 2011; Wald et al., 2011
*gD2 subunit vaccine*	GlaxoSmithKline	gD2 with AS04 (dMPL)	Completed Phase III (DC)	Keadle et al., 1997; Belshe et al., 2012;^41,2^
*G103*	Immune Design	HSV-2 gD, deletions in UL19 and UL25+GLA-SE adjuvant	Phase I ongoing	Odegard et al., 2016
*Replication Defective Viral Vaccines*				
*CJ2-gD2*		Non-replicating gD2 dominant neg HSV-2	Pre-Clinical	Zhang et al., 2014
*HSV529*	Sanofi Pasteur	Replication Defective HSV-1, UL5, UL29 deletion	Phase I	Bernard et al., 2015; Dropulic et al., 2019; Wang et al., 2021
*HSV-1 vhs-/ICP8-*		Deletions in Vhs and ICP8	Pre-Clinical	Geiss et al., 2000; Keadle et al., 2002
*DNA Vaccines*				
*COR-1*	Admedus	gD2 ubiquitin tag, codon optimized DNA vaccine	Phase II	Dutton et al., 2013; Chandra et al., 2019
*pRSC-gD-IL-2123*	Southeast University, China	HSV-1 gD combined with IL-21	Pre-clinical	Hu et al., 2011
*VCL-HB01/HM01*	Vical	DNA vaccine: gD2+/−UL46/Vaxfectin	Phase II (DC)	Shlapobersky et al., 2012; Veselenak et al., 2012; Riedmann, 2014
*gB1s-NISV*		Intranasal non-ionic surfactant vesicles containing recombinant HSV-1 gB+CpG	Pre-Clinical	Cortesi et al., 2013

### Live-Attenuated Vaccines

Various live-attenuated vaccines for HSV have been tested in the pre-clinical and the clinical stages. While live-attenuated vaccines are particularly effective at mounting a humoral and cell-mediated immune response, one of the primary challenges of the live-attenuated vaccine remains vaccine safety.

The R2 vaccine is a live-attenuated HSV-1 strain encoding pUL37 tegument protein with mutations in region 2, which is critical for nervous system invasion and the establishment of latency. Viral protection was assessed with vaginal HSV-2 challenge in guinea pig genital model. Results showed decreased severity of HSV-2 disease, both acute and recurrent forms. Viral shedding during recurrence decreased by 33–64% in each of the groups vaccinated with R2, with superior results to a subunit gD2+MPL/Alum vaccine (Bernstein et al., 2020).

The HSV-2 ΔgD-2 vaccine is a single cycle virus with deletion in glycoprotein D has been shown in previous murine model studies to be protective against ten times the lethal dose of HSV-1 or HSV-2, without detection of latent virus (Burn et al., 2018). Protection conferred by this vaccine was shown to be mediated by antibody-dependent cell-mediated cytotoxicity (ADCC). Passive transfer studies showed antibodies that activated murine Fc receptors to activate ADCC and phagocytosis (Petro et al., 2016).

Recent studies in a murine model have shown that the ΔgD-2 vaccine prevented the latent infection in the sacral ganglion following HSV-2 challenge in HSV-1 seronegative and seropositive mice. However, it did not have a discernible effect on the size of the HSV-1 latency in the trigeminal ganglia. It was compared to a subunit rgD2/alum-MPL vaccine – which did not elicit the same response – increased total HSV-specific antibodies and ADCC responses and protection from a high dose lethal HSV-2 challenge (Burn Aschner et al., 2020).

### Subunit Vaccines

Subunit vaccines have become a powerful tool to mount immune responses against viral proteins. Particularly, the success of Shingrix to mount an immune response again Herpes Zoster has increased interest to develop to subunit vaccinations for herpesvirus (Heineman et al., 2019). While subunit vaccinations are safer than live-attenuated vaccines, the challenge with the development of these vaccinations lies with generating an effective, long-lasting immune response. Subunit vaccines are often administered with adjuvants for this reason (Karch and Burkhard, 2016).

The glycoprotein D2 vaccine by GlaxoSmithKline is a subunit vaccine consisting of the HSV-2 glycoprotein D (gD2-AS04) with adjuvants including aluminum hydroxide and 3-O-deacylated monophosphoryl lipid A (MPL). gD2 is required for viral entry into cells. Clinical trial data (Herpevac) were observed to induce significant protection against genital HSV-1 infection and disease, but not disease or infection with HSV-2. HSV-2 and HSV-1 gD amino acids show 89% homology. Variance in protein structure was attributed to the different effects (Belshe et al., 2012). Results further showed 3.5-fold higher neutralizing antibody titers to HSV-1 compared to HSV-2. Shielding neutralizing domains (gC2 and gE2 of the HSV-2 virus) was proposed as a potential mechanism for the varied response. This is clinically significant because HSV-1 has emerged as the leading cause of primary genital disease (Awasthi et al., 2014).

HERPV is a subunit vaccine that consists of 32 HSV-2 peptides originating from 22 HSV-2 proteins, complexed with HSP70 chaperone protein with a QS21 saponin adjuvant. Previous studies with mice prophylaxis model and guinea pigs therapeutic model showed protection from viral challenge. Recent clinical trial data from HSV-2^+^ participants treated with the vaccine showed mononuclear cell reactivity and CD8^+^ T-cell expansion (Mo et al., 2011; Wald et al., 2011).

GEN-003 is a subunit vaccine with a transmembrane deletion mutant of glycoprotein D (gD2ΔTMR), a fragment of infected cell protein 4 (ICP4.2) and Matrix-M2, a saponin-derived adjuvant. GEN-003 was demonstrated to be safe in recent Clinical Trials Gov. (2020), reducing genital HSV-2 shedding and lesion rates. It was also shown to stimulate both humoral and cellular responses, with neutralizing antibodies and T-cell responses (measured with gamma interferon ELISPOT; Bernstein et al., 2017). Immunization also resulted in significant decreases in viral shedding and the rate of lesions for 1year (Van Wagoner et al., 2018).

The trivalent vaccine consists of glycoproteins C, D, and E with a CpG/aluminum adjuvant. Glycoproteins C and E function as immune evasion molecules. Studies in rhesus macaques have shown that the trivalent vaccine is able to induce neutralizing antibodies (mucosa and plasma), antibodies obstructing gC2 and gE2 immune evasion activity. It additionally stimulated CD4^+^ T-cell responses. Vaccine efficacy was tested in the guinea pig model and was shown to be very efficacious in reducing the duration of genital lesions and decreasing lesion incidence (Awasthi et al., 2017).

G103 is formulated with recombinant HSV-2 proteins gD, UL19 and UL25 gene products with TLR4 agonist glucopyranosyl lipid A (GLA) adjuvant. Prophylactic vaccination in the murine model showed complete protection against lethal HSV-2 infection, with transient replication in the genital mucosa and sterilizing immunity in the dorsal root ganglion. The vaccine was also shown to expand CD4 and CD8 T cells in HSV-2^+^-infected mice as well. In a guinea pig model, therapeutic vaccination was 50% effective at reducing the number of lesions per subject as well as the total lesions in the treatment group (Odegard et al., 2016).

### Nucleic Acid Vaccines (DNA/mRNA)

Nucleic acid vaccines mainly consist of DNA plasmid vaccines and mRNA vaccines. The purpose of these vaccines is to transfer genetic material to cells in order to express and produce proteins. These proteins serve as antigens for the human immune system.

Vaccine research has mostly focused on the development of DNA vaccines over mRNA given the difficulties involving mRNA stability and creating an effective delivery system. However, recently mRNA technology has evolved significantly, with significant advantages. They do not integrate into the host genome, can be translated in proliferating and non-proliferating cells, and show immediate protein production for a controllable amount of time (Pardi et al., 2018).

COR-1 is a DNA vaccine with two codon-modified and optimized plasmids, one coding for the HSV-2 envelope glycoprotein D (gD2) and the second with a truncated gD2 fused with ubiquitin. This vaccine was tested in a murine model, inducing both cellular and humoral responses, with protection from lethal virus challenge and reduced viral latency (Dutton et al., 2013). Clinical trial data on HSV-2-infected subjects demonstrated safety of the vaccine in humans and reduced viral shedding after vaccine administration (Chandra et al., 2019).

A new formulation of the trivalent vaccine with HSV-2 glycoproteins C, D, and E as an mRNA vaccine has recently been studied in the murine model. This study compared the new mRNA formulation with the existing subunit vaccine. The mRNA vaccine developed is a nucleoside-modified mRNA molecule in lipid nanoparticles (LNP). In the murine and guinea pig model, the mRNA-LNP vaccine yielded a superior humoral response in comparison with the subunit vaccine, with higher titers of neutralizing antibodies, and antibodies for gD2 epitopes associated with cellular entry. Additionally, the mRNA vaccine produced superior cell-mediated responses compared to the subunit vaccine, with improved CD4^+^ T-cell responses including T-follicular CD4^+^ (Tfh) responses and germinal center B-cell responses. While both formulations were effective and preventing genital lesions in mice and guinea pigs, the mRNA vaccine was superior at preventing HSV-2 infection of the dorsal root ganglia and had reduced viral shedding (Awasthi et al., 2019). Studies have also shown that this HSV-2 mRNA vaccine outperforms the subunit vaccine in protecting against HSV-1 genital infection. It prevented invasion of the dorsal root ganglion (Egan et al., 2020).

### Replication Defective Virus Vaccine

Dl5-29 (HSV529) is a strain of HSV-2 with mutations in essential viral genes UL5 and UL29 making it replication defective. It was tested as both a prophylactic and therapeutic vaccine. It was shown to be safe, producing neutralizing antibodies and CD4^+^ T-cell responses in seronegative subjects who were vaccinated (Dropulic et al., 2019). Recent studies have demonstrated the production of antibodies mediating NK cell activation. Additionally, HSV-2 gD antibodies were detected in cervicovaginal fluid at around one-third of the serum level (Wang et al., 2021).

## Approach To Herpes Simplex Virus Therapy

Nucleoside analogs, including acyclovir, valacyclovir, and famciclovir, remain standard therapies for mucocutaneous and visceral HSV infection. Idoxuridine, trifluorothymidine, vidarabine, and cidofovir are used topically for ocular HSV infections (Beigel and Kottilil, 2020).

Development of HSV resistance to acyclovir and valacyclovir is rare despite extensive use for treatment of infection (prevalence ~1%). Increased prevalence is seen in patients with herpetic keratitis (prevalence of up to 7%; Burrel et al., 2020). Antiviral resistance is increased in immunocompromised patients, specifically patients with HIV infections (5% prevalence) and bone marrow transplants (prevalence up to 30%; Beigel and Kottilil, 2020). IV Foscarnet and cidofovir are usually effective for acyclovir resistant viral strains (Beigel and Kottilil, 2020). Continued exposure to cidofovir does not easily induce resistance. However, there have been case reports of cidofovir-resistant HSV and CMV (Wyles et al., 2005).

## Discussion

There have been various attempts at formulating an effective vaccine for herpes simplex virus, especially given its wide prevalence and ability to cause significant morbidity and mortality.

A prophylactic vaccine would be ideal. It would be effective at preventing active infection and transmission of the virus, which would avoid latent infection of the dorsal root ganglia (trigeminal and sacral ganglia), reactivation and the clinical manifestations accompanying it. This would prevent the sequelae of primary infection and viral spread within the population and severe complications involving reactivation. However, the utility of such a vaccine is in question. Most herpesvirus infections occur in adolescence (James et al., 2020), so any prophylactic vaccine would only have optimal utility if it was safe for administration during early childhood.

While a prophylactic vaccine would be optimal for the prevention of all complications, it might be more realistic to focus on therapeutic vaccines that would reduce disease severity (measured in recurrences, duration of clinical symptoms and viral shedding). While most therapeutic vaccines are not as effective at targeting latent virus, recent work in genome editing involving homing endonucleases and CRISPR/cas9 systems offer a bridge to complete viral clearance (Zhang et al., 2021).

A therapeutic vaccine would potentially be able to benefit a wider proportion of the populations given the high proportion of seropositive individuals (James et al., 2020). HSV infection has a wide range of presentation that varies by the individual (ranging from asymptomatic to severe complications like ocular keratitis; Bolognia et al., 2018). A therapeutic vaccine would be cost-effective and more efficiently administered compared to a prophylactic vaccine, targeting the subset of seropositive individuals with clinical symptoms. This would be a more effective strategy for the prevention of severe clinical complications compared to prophylactic vaccines. While there are risk factors for primary infection with HSV, there are no clear risk factors for the development of severe complications which would make it difficult to target vulnerable populations only, instead of the entire population. What has emerged with the development of vaccines for herpes is some vaccines like the G103 vaccine with prophylactic as well as a therapeutic utility (Odegard et al., 2016).

HSV-1 and HSV-2 show 50% homology (Berger and Houff, 2008) of their genome, with coinfection yielding interspecies recombinants (Davison, 2007; Koelle et al., 2017). Although they are with their differences in molecular structure, the vast majority of vaccine candidates (while targeted to one subtype) have been shown to be effective in both HSV-1 and HSV-2 models. This is especially reassuring since an increasing proportion of the population (about 50%) has genital infection from HSV-1, as opposed to HSV-2 which used to be the dominant strain (Whitley and Green, 2019). Furthermore, this cross-protection would be useful to prevent not only the genital reactivations of herpesvirus, but the ocular and systemic manifestations as well.

Current therapies for HSV including acyclovir and valacyclovir are effective at reducing viral shedding, symptom duration, and severity (Gupta et al., 2004). However, there is a short treatment window for effective treatment. Treatment needs to be started in the prodromal phase to have optimal effects on controlling viral replication, as late treatment has limited effectiveness. Additionally, their effectiveness is limited with only marginal reductions to symptom durations and severity (Harmenberg et al., 2010). Suppressive treatment is not effective at the prevention and reduction of reactivation events, and transmission is reduced by only 50% (Birkmann and Zimmermann, 2016).

Given our knowledge of HSV pathogenesis and latency, an immune system modifying drug or therapeutic vaccination has potential to address these limitations. Although the prevalence of acyclovir-, valacyclovir,- and cidofovir-resistant strains of HSV is low, therapeutic vaccination has an additional use as an alternative treatment for those rare cases (Beigel and Kottilil, 2020). This is particularly the case with increasing resistance to acyclovir in immunocompromised patients, where IV Foscarnet, the next-line treatment, has limitations due to its side effects (Harmenberg et al., 2010).

Taking into account the increased incidence of resistance in immunocompromised patients, it would be prudent to prioritize the development of subunit, nucleic acid vaccines, and replication defective vaccines over live-attenuated vaccines (Birkmann and Zimmermann, 2016). Live-attenuated vaccines, while effective, are associated with increased safety concerns, especially in immunocompromised patients.

### Further Considerations for the Future of Herpes Simplex Virus Vaccine Development

Our understanding of the complexities of herpes simplex virus pathogenesis and immune evasion is consistently evolving, along with our understanding of viral latency. Designing an effective therapeutic or prophylactic vaccine requires further understanding these processes.

Trials that had been successful in the pre-clinical realm with murine model and guinea pig models have been effective, but have not translated well in the clinical realm. Other trials like the GSK gD subunit vaccine and the GEN-003 vaccine have been suspended due to dwindling financial support (Awasthi et al., 2014; Genocea Biosciences, 2018). To us, the HSV-529 and G103 appear to be promising from early studies in the pre-clinical stage. Additionally, they are likely to have less issues with safety as they are not live-attenuated vaccines.

The rapid development of an mRNA vaccine for COVID-19 has re-vitalized interest in mRNA vaccines as viable options to produce viral immunity. The classical problems involving the creation of mRNA vaccines include mRNA stability and delivery systems. It remains to be seen whether mRNA vaccines might have a utility for HSV; however, initial studies with the trivalent vaccine have been promising, showing increased efficacy compared to a subunit formulation (Liu et al., 2021).

The advantages of an mRNA vaccine were previously discussed: It does not integrate within the host genome, translates in both proliferating and non-proliferating cells, with immediate protein production for a controllable amount of time (Pardi et al., 2018).

It would additionally be prudent to utilize our increasing knowledge of the pathogenesis of herpes simplex virus and its interaction with the immune system in order to formulate novel therapies that could include therapeutic vaccination.

## Author Contributions

PS contributed to conception and outline of the article. RK wrote the first draft of the manuscript. All authors contributed to manuscript revision, read, and approved the submitted version.

## Funding

This work was supported by National Institutes of Health Grants EY16352 (PS), EY21247 (PS) and an unrestricted grant from Research to Prevent Blindness to the Department of Ophthalmology, Saint Louis University.

## Conflict of Interest

The authors declare that the research was conducted in the absence of any commercial or financial relationships that could be construed as a potential conflict of interest.

## Publisher’s Note

All claims expressed in this article are solely those of the authors and do not necessarily represent those of their affiliated organizations, or those of the publisher, the editors and the reviewers. Any product that may be evaluated in this article, or claim that may be made by its manufacturer, is not guaranteed or endorsed by the publisher.

## References

[1] Alberts B. Johnson A. Lewis J. Raff M. Roberts K. Walter P. (2002). Molecular Biology of the Cell. 4th *Edn*. New York: Garland Science.

[2] Awasthi S. Belshe R. B. Friedman H. M. (2014). Better neutralization of herpes simplex virus type 1 (HSV-1) than HSV-2 by antibody from recipients of GlaxoSmithKline HSV-2 glycoprotein D2 subunit vaccine. J. Infect. Dis. 210, 571–575. doi: 10.1093/infdis/jiu177, PMID: 24652496 PMC4172040

[3] Awasthi S. Hook L. M. Pardi N. Wang F. Myles A. Cancro M. P.. (2019). Nucleoside-modified mRNA encoding HSV-2 glycoproteins C, D, and E prevents clinical and subclinical genital herpes. Sci. Immunol. 4:eaaw7083. doi: 10.1126/sciimmunol.aaw7083, PMID: 31541030 PMC6822172

[4] Awasthi S. Hook L. M. Shaw C. E. Pahar B. Stagray J. A. Liu D.. (2017). An HSV-2 trivalent vaccine is immunogenic in rhesus macaques and highly efficacious in Guinea pigs. PLoS Pathog. 13:e1006141. doi: 10.1371/journal.ppat.1006141, PMID: 28103319 PMC5245903

[5] Awasthi S. Lubinski J. M. Shaw C. E. Barrett S. M. Cai M. Wang F.. (2011). Immunization with a vaccine combining herpes simplex virus 2 (HSV-2) glycoprotein C (gC) and gD subunits improves the protection of dorsal root ganglia in mice and reduces the frequency of recurrent vaginal shedding of HSV-2 DNA in Guinea pigs compared to immunization with gD alone. J. Virol. 85, 10472–10486. doi: 10.1128/JVI.00849-11, PMID: 21813597 PMC3187515

[6] Beigel J. H. Kottilil S. (2020). “Antiviral therapy (Non-HIV).” in Goldman-Cecil Medicine. eds. L. Goldman A. I. Schafer (Netherlands: Elsevier Press), 2140–2150.

[7] Belshe R. B. Leone P. A. Bernstein D. I. Wald A. Levin M. J. Stapleton J. T.. (2012). Efficacy results of a trial of a herpes simplex vaccine. N. Engl. J. Med. 366, 34–43. doi: 10.1056/NEJMoa1103151, PMID: 22216840 PMC3287348

[8] Berger J. R. Houff S. (2008). Neurological complications of herpes simplex virus type 2 infection. Arch. Neurol. 65, 596–600. doi: 10.1001/archneur.65.5.596, PMID: 18474734

[9] Bernard M. C. Barban V. Pradezynski F. de Montfort A. Ryall R. Caillet C.. (2015). Immunogenicity, protective efficacy, and non-replicative status of the HSV-2 vaccine candidate HSV529 in mice and Guinea pigs. PLoS One 10:e0121518. doi: 10.1371/journal.pone.0121518, PMID: 25837802 PMC4383384

[10] Bernstein D. I. Cardin R. D. Smith G. A. Pickard G. E. Sollars P. E. Dixon D. A.. (2020). The R2 non-neuroinvasive HSV-1 vaccine affords protection from genital HSV-2 infections in a Guinea pig model. NPJ Vacc. 5:104. doi: 10.1038/s41541-020-00254-8, PMID: 33298966 PMC7648054

[11] Bernstein D. I. Flechtner J. B. McNeil L. K. Heineman T. Oliphant T. Tasker S.. (2019). Therapeutic HSV-2 vaccine decreases recurrent virus shedding and recurrent genital herpes disease. Vaccine 37, 3443–3450. doi: 10.1016/j.vaccine.2019.05.009, PMID: 31103365

[12] Bernstein D. I. Wald A. Warren T. Fife K. Tyring S. Lee P.. (2017). Therapeutic vaccine for genital herpes simplex Virus-2 infection: findings From a randomized trial. J. Infect. Dis. 215, 856–864. doi: 10.1093/infdis/jix004, PMID: 28329211 PMC7206854

[13] Birkmann A. Zimmermann H. (2016). HSV antivirals - current and future treatment options. Curr. Opin. Virol. 18, 9–13. doi: 10.1016/j.coviro.2016.01.013, PMID: 26897058

[14] BlueWillow Biologics (2019). BlueWillow Biologics Awarded Patent for Intranasal Genital Herpes Vaccine. https://bluewillow.com/bluewillow-biologics-awarded-patent-for-intranasal-genital-herpes-vaccine/ (Accessed September 30, 2021)

[15] Bolognia J. L. Schaffer J. V. Cerroni L. (2018). “Human herpesviruses.” in Dermatology. 4th *Edn*. eds. J. P. Callen E. W. Cowen G. J. Hruza J. I. Jorizzo H. Lui L. Requena. (Netherlands: Elsevier).

[16] Burn Aschner C. Knipe D. M. Herold B. C. (2020). Model of vaccine efficacy against HSV-2 superinfection of HSV-1 seropositive mice demonstrates protection by antibodies mediating cellular cytotoxicity. NPJ vaccines 5:35. doi: 10.1038/s41541-020-0184-7, PMID: 32411398 PMC7206093

[17] Burn C. Ramsey N. Garforth S. J. Almo S. Jacobs W. R. Jr. Herold B. C. (2018). A herpes simplex virus (HSV)-2 single-cycle candidate vaccine deleted in glycoprotein D protects male mice From lethal skin challenge With clinical isolates of HSV-1 and HSV-2. J. Infect. Dis. 217, 754–758. doi: 10.1093/infdis/jix628, PMID: 29216362 PMC5853290

[18] Burrel S. Topalis D. Boutolleau D. (2020). Résistance des virus herpes simplex aux antiviraux [Herpes simplex virus resistance to antivirals]. Virologie 24, 325–342. doi: 10.1684/vir.2020.0864 33111706

[19] Chandra J. Woo W. P. Dutton J. L. Xu Y. Li B. Kinrade S.. (2019). Immune responses to a HSV-2 polynucleotide immunotherapy COR-1 in HSV-2 positive subjects: A randomized double blinded phase I/IIa trial. PLoS One 14:e0226320. doi: 10.1371/journal.pone.0226320, PMID: 31846475 PMC6917347

[20] Chiuppesi F. Vannucci L. De Luca A. Lai M. Matteoli B. Freer G.. (2012). A lentiviral vector-based, herpes simplex virus 1 (HSV-1) glycoprotein B vaccine affords cross-protection against HSV-1 and HSV-2 genital infections. J. Virol. 86, 6563–6574. doi: 10.1128/JVI.00302-12, PMID: 22491465 PMC3393530

[21] Clinical Trials Gov. (2020). Safety and Efficacy of 4 Investigational HSV 2 Vaccines in Adults With Recurrent Genital Herpes Caused by HSV 2 (HSV15). Available at: https://clinicaltrials.gov/ct2/show/NCT04222985 (Accessed September 30, 2021)

[22] Cortesi R. Ravani L. Rinaldi F. Marconi P. Drechsler M. Manservigi M.. (2013). Intranasal immunization in mice with non-ionic surfactants vesicles containing HSV immunogens: a preliminary study as possible vaccine against genital herpes. Int. J. Pharm. 440, 229–237. doi: 10.1016/j.ijpharm.2012.06.042, PMID: 22743007

[23] Davison A. J. (2007). “Comparative Analysis of the Genomes.” in Human Herpesviruses: Biology, Therapy, and Immunoprophylaxis. eds. Arvin A. Campadelli-Fiume G. Mocarski E. (Cambridge: Cambridge University Press) 21348071

[24] Denes C. E. Everett R. D. Diefenbach R. J. (2020). Tour de herpes: cycling Through the life and biology of HSV-1. Methods Mol. Biol. 2060, 1–30. doi: 10.1007/978-1-4939-9814-2_1 31617170

[25] Dolan A. Jamieson F. E. Cunningham C. Barnett B. C. McGeoch D. J. (1998). The genome sequence of herpes simplex virus type 2. J. Virol. 72, 2010–2021. doi: 10.1128/JVI.72.3.2010-2021.1998 9499055 PMC109494

[26] Dropulic L. K. Oestreich M. C. Pietz H. L. Laing K. J. Hunsberger S. Lumbard K.. (2019). A randomized, double-blinded, placebo-controlled, phase 1 study of a replication-defective herpes simplex virus (HSV) type 2 vaccine, HSV529, in adults With or Without HSV infection. J. Infect. Dis. 220, 990–1000. doi: 10.1093/infdis/jiz225, PMID: 31058977 PMC6688060

[27] Dutton J. L. Li B. Woo W. P. Marshak J. O. Xu Y. Huang M. L.. (2013). A novel DNA vaccine technology conveying protection against a lethal herpes simplex viral challenge in mice. PLoS One 8:e76407. doi: 10.1371/journal.pone.0076407, PMID: 24098493 PMC3789751

[28] Egan K. Hook L. M. Naughton A. Friedman H. M. Awasthi S. (2020). Herpes simplex virus type 2 trivalent protein vaccine containing glycoproteins C, D and E protects Guinea pigs against HSV-1 genital infection. Hum. Vacc. Immuno. 16, 2109–2113. doi: 10.1080/21645515.2020.1749509, PMID: 32347775 PMC7553673

[29] English L. Chemali M. Duron J. Rondeau C. Laplante A. Gingras D.. (2009). Autophagy enhances the presentation of endogenous viral antigens on MHC class I molecules during HSV-1 infection. Nat. Immunol. 10, 480–487. doi: 10.1038/ni.1720, PMID: 19305394 PMC3885169

[30] Flechtner J. B. Long D. Larson S. Clemens V. Baccari A. Kien L.. (2016). Immune responses elicited by the GEN-003 candidate HSV-2 therapeutic vaccine in a randomized controlled dose-ranging phase 1/2a trial. Vaccine 34, 5314–5320. doi: 10.1016/j.vaccine.2016.09.001, PMID: 27642130

[31] Geiss B. J. Smith T. J. Leib D. A. Morrison L. A. (2000). Disruption of virion host shutoff activity improves the immunogenicity and protective capacity of a replication-incompetent herpes simplex virus type 1 vaccine strain. J. Virol. 74, 11137–11144. doi: 10.1128/jvi.74.23.11137-11144.2000, PMID: 11070010 PMC113198

[32] Genocea Biosciences (2018). Genocea Reports Fourth Quarter and Full-Year 2017 Financial Results. Available at: https://www.globenewswire.com/news-release/2018/02/15/1348763/0/en/Genocea-Reports-Fourth-Quarter-and-Full-Year-2017-Financial-Results.html (Accessed September 2021)

[33] Gupta R. Wald A. Krantz E. Selke S. Warren T. Vargas-Cortes M.. (2004). Valacyclovir and acyclovir for suppression of shedding of herpes simplex virus in the genital tract. J. Infect. Dis. 190, 1374–1381. doi: 10.1086/424519, PMID: 15378428

[34] Hadar T. Sarov I. (1984). Specific IgG and IgA antibodies to herpes simplex virus (HSV)-induced surface antigen in patients with HSV infections and in healthy adults. J. Med. Virol. 14, 201–207. doi: 10.1002/jmv.1890140303, PMID: 6094720

[35] Halford W. P. Püschel R. Gershburg E. Wilber A. Gershburg S. Rakowski B. (2011). A live-attenuated HSV-2 ICP0 virus elicits 10 to 100 times greater protection against genital herpes than a glycoprotein D subunit vaccine. PLoS One 6:e17748. doi: 10.1371/journal.pone.0017748, PMID: 21412438 PMC3055896

[36] Halford W. P. Püschel R. Rakowski B. (2010). Herpes simplex virus 2 ICP0 mutant viruses are avirulent and immunogenic: implications for a genital herpes vaccine. PLoS One 5:e12251. doi: 10.1371/journal.pone.0012251, PMID: 20808928 PMC2923193

[37] Harmenberg J. Oberg B. Spruance S. (2010). Prevention of ulcerative lesions by episodic treatment of recurrent herpes labialis: A literature review. Acta Derm. Venereol. 90, 122–130. doi: 10.2340/00015555-0806, PMID: 20169294

[38] Heineman T. C. Cunningham A. Levin M. (2019). Understanding the immunology of Shingrix, a recombinant glycoprotein E adjuvanted herpes zoster vaccine. Curr. Opin. Immunol. 59, 42–48. doi: 10.1016/j.coi.2019.02.009, PMID: 31003070

[39] Hook L. M. Awasthi S. Dubin J. Flechtner J. Long D. Friedman H. M. (2019). A trivalent gC2/gD2/gE2 vaccine for herpes simplex virus generates antibody responses that block immune evasion domains on gC2 better than natural infection. Vaccine 37, 664–669. doi: 10.1016/j.vaccine.2018.11.076, PMID: 30551986 PMC6447314

[40] Hook L. M. Lubinski J. M. Jiang M. Pangburn M. K. Friedman H. M. (2006). Herpes simplex virus type 1 and 2 glycoprotein C prevents complement-mediated neutralization induced by natural immunoglobulin M antibody. J. Virol. 80, 4038–4046. doi: 10.1128/JVI.80.8.4038-4046.2006, PMID: 16571820 PMC1440426

[41] HSV-040 Study Group Abu-Elyazeed R. R. Heineman T. Dubin G. Fourneau M. Leroux-Roels I. (2013). Safety and immunogenicity of a glycoprotein D genital herpes vaccine in healthy girls 10-17 years of age: results from a randomised, controlled, double-blind trial. Vaccine 31, 6136–6143. doi: 10.1016/j.vaccine.2013.06.081, PMID: 23850416

[42] Hu K. Dou J. Yu F. He X. Yuan X. Wang Y.. (2011). An ocular mucosal administration of nanoparticles containing DNA vaccine pRSC-gD-IL-21 confers protection against mucosal challenge with herpes simplex virus type 1 in mice. Vaccine 29, 1455–1462. doi: 10.1016/j.vaccine.2010.12.031, PMID: 21185849

[43] James C. Harfouche M. Welton N. J. Turner K. M. Abu-Raddad L. J. Gottlieb S. L.. (2020). Herpes simplex virus: global infection prevalence and incidence estimates, 2016. Bull. World Health Organ. 98, 315–329. doi: 10.2471/BLT.19.237149, PMID: 32514197 PMC7265941

[44] Jiao X. Sui H. Lyons C. Tran B. Sherman B. T. Imamichi T. (2019). Complete genome sequence of herpes simplex virus 1 strain McKrae. Microbiol. Res. Announce. 8:e00993-19. doi: 10.1128/MRA.00993-19, PMID: 31558635 PMC6763650

[45] Johnston C. Gottlieb S. L. Wald A. (2016). Status of vaccine research and development of vaccines for herpes simplex virus. Vaccine 34, 2948–2952. doi: 10.1016/j.vaccine.2015.12.076, PMID: 26973067

[46] Jouan Y. Grammatico-Guillon L. Espitalier F. Cazals X. François P. Guillon A. (2015). Long-term outcome of severe herpes simplex encephalitis: a population-based observational study. Crit. Care 19:345. doi: 10.1186/s13054-015-1046-y, PMID: 26387515 PMC4576407

[47] Karch C. P. Burkhard P. (2016). Vaccine technologies: From whole organisms to rationally designed protein assemblies. Biochem. Pharmacol. 120, 1–14. doi: 10.1016/j.bcp.2016.05.001, PMID: 27157411 PMC5079805

[48] Keadle T. L. Laycock K. A. Miller J. K. Hook K. K. Fenoglio E. D. Francotte M.. (1997). Efficacy of a recombinant glycoprotein D subunit vaccine on the development of primary and recurrent ocular infection with herpes simplex virus type 1 in mice. J. Infec. Dis. 176, 331–338. doi: 10.1086/514049, PMID: 9237697

[49] Keadle T. L. Morrison L. A. Morris J. L. Pepose J. S. Stuart P. M. (2002). Therapeutic immunization with a virion host shutoff (vhs) defective, replication-incompetent HSV-1 strain limits recurrent herpetic ocular infection. J. Virol. 76, 3615–3625. doi: 10.1128/jvi.76.8.3615-3625.2002, PMID: 11907201 PMC136075

[50] Koelle D. M. Norberg P. Fitzgibbon M. P. Russell R. M. Greninger A. L. Huang M. L.. (2017). Worldwide circulation of HSV-2×HSV-1 recombinant strains. Sci. Rep. 7:44084. doi: 10.1038/srep44084, PMID: 28287142 PMC5347006

[51] Leib D. A. Alexander D. E. Cox D. Yin J. Ferguson T. A. (2009). Interaction of ICP34.5 with Beclin 1 modulates herpes simplex virus type 1 pathogenesis through control of CD4+ T-cell responses. J. Virol. 83, 12164–12171. doi: 10.1128/JVI.01676-09, PMID: 19759141 PMC2786728

[52] Lim F. Khalique H. Ventosa M. Baldo A. (2013). Biosafety of gene therapy vectors derived from herpes simplex virus type 1. Curr. Gene Ther. 13, 478–491. doi: 10.2174/156652321306140103224550, PMID: 24397529

[53] Liu Z. Guan Y. Sun X. Shi L. Liang R. Lv X.. (2013). HSV-1 activates NF-kappaB in mouse astrocytes and increases TNF-alpha and IL-6 expression via toll-like receptor 3. Neurol. Res. 35, 755–762. doi: 10.1179/016164113X13703372991516, PMID: 23947625

[54] Liu T. Liang Y. Huang L. (2021). Development and delivery systems of mRNA vaccines. Front. Bioeng. Biotechnol. 9:718753. doi: 10.3389/fbioe.2021.718753, PMID: 34386486 PMC8354200

[55] Lund J. Sato A. Akira S. Medzhitov R. Iwasaki A. (2003). Toll-like receptor 9-mediated recognition of herpes simplex virus-2 by plasmacytoid dendritic cells. J. Exp. Med. 198, 513–520. doi: 10.1084/jem.20030162, PMID: 12900525 PMC2194085

[56] Madavaraju K. Koganti R. Volety I. Yadavalli T. Shukla D. (2021). Herpes simplex virus cell entry mechanisms: An update. Front. Cell. Infect. Microbiol. 10:617578. doi: 10.3389/fcimb.2020.617578, PMID: 33537244 PMC7848091

[57] Maruzuru Y. Ichinohe T. Sato R. Miyake K. Okano T. Suzuki T.. (2018). Herpes simplex virus 1 VP22 inhibits AIM2-dependent Inflammasome activation to enable efficient viral replication. Cell Host Microbe 23, 254–265. doi: 10.1016/j.chom.2017.12.014, PMID: 29447697

[58] Mo A. Musselli C. Chen H. Pappas J. Leclair K. Liu A.. (2011). A heat shock protein based polyvalent vaccine targeting HSV-2: CD4(+) and CD8(+) cellular immunity and protective efficacy. Vaccine 29, 8530–8541. doi: 10.1016/j.vaccine.2011.07.011, PMID: 21767588

[59] Odegard J. M. Flynn P. A. Campbell D. J. Robbins S. H. Dong L. Wang K.. (2016). A novel HSV-2 subunit vaccine induces GLA-dependent CD4 and CD8 T cell responses and protective immunity in mice and Guinea pigs. Vaccine 34, 101–109. doi: 10.1016/j.vaccine.2015.10.137, PMID: 26571309 PMC6322202

[60] Pardi N. Hogan M. J. Naradikian M. S. Parkhouse K. Cain D. W. Jones L.. (2018). Nucleoside-modified mRNA vaccines induce potent T follicular helper and germinal center B cell responses. J. Exp. Med. 215, 1571–1588. doi: 10.1084/jem.20171450, PMID: 29739835 PMC5987916

[61] Petro C. González P. A. Cheshenko N. Jandl T. Khajoueinejad N. Bénard A.. (2015). Herpes simplex type 2 virus deleted in glycoprotein D protects against vaginal, skin and neural disease. Elife 4:e06054. doi: 10.7554/eLife.06054, PMID: 25756612 PMC4352706

[62] Petro C. D. Weinrick B. Khajoueinejad N. Burn C. Sellers R. Jacobs W. R.. (2016). HSV-2 ΔgD elicits FcγR-effector antibodies that protect against clinical isolates. JCI Insight 1:e88529. doi: 10.1172/jci.insight.88529, PMID: 27536733 PMC4985247

[63] Piret J. Boivin G. (2016). Antiviral resistance in herpes simplex virus and varicella-zoster virus infections: diagnosis and management. Curr. Opin. Infect. Dis. 29, 654–662. doi: 10.1097/QCO.0000000000000288, PMID: 27306564

[64] Prichard M. N. Kaiwar R. Jackman W. T. Quenelle D. C. Collins D. J. Kern E. R.. (2005). Evaluation of AD472, a live attenuated recombinant herpes simplex virus type 2 vaccine in Guinea pigs. Vaccine 23, 5424–5431. doi: 10.1016/j.vaccine.2005.02.028, PMID: 15950327 PMC2718572

[65] Richards A. L. Sollars P. J. Pitts J. D. Stults A. M. Heldwein E. E. Pickard G. E.. (2017). The pUL37 tegument protein guides alpha-herpesvirus retrograde axonal transport to promote neuroinvasion. PLoS Pathog. 13:e1006741. doi: 10.1371/journal.ppat.1006741, PMID: 29216315 PMC5749899

[66] Riedmann E. M. (2014). Vical initiates vaccine trials against HSV-2 and CMV. Hum. Vacc. Immuno. 10:255. doi: 10.4161/hv.29344 PMID: 24963522

[67] Russell R. G. Nasisse M. P. Larsen H. S. Rouse B. T. (1984). Role of T-lymphocytes in the pathogenesis of herpetic stromal keratitis. Invest. Ophthalmol. Vis. Sci. 8, 938–944. PMID: 6611324

[68] Schiffer J. T. Abu-Raddad L. Mark K. E. Zhu J. Selke S. Koelle D. M.. (2010). Mucosal host immune response predicts the severity and duration of herpes simplex virus-2 genital tract shedding episodes. Proc. Natl. Acad. Sci. U. S. A. 107, 18973–18978. doi: 10.1073/pnas.1006614107, PMID: 20956313 PMC2973882

[69] Sela M. Hilleman M. R. (2004). Therapeutic vaccines: realities of today and hopes for tomorrow. Proc. Natl. Acad. Sci. U. S. A., 101, (Supplement 2):14559. doi: 10.1073/pnas.0405924101, PMID: 15383664 PMC522000

[70] Shlapobersky M. Marshak J. O. Dong L. Huang M. L. Wei Q. Chu A.. (2012). Vaxfectin-adjuvanted plasmid DNA vaccine improves protection and immunogenicity in a murine model of genital herpes infection. J. Gen. Virol. 93, 1305–1315. doi: 10.1099/vir.0.040055-0, PMID: 22398318 PMC3755514

[71] Skoberne M. Cardin R. Lee A. Kazimirova A. Zielinski V. Garvie D.. (2013). An adjuvanted herpes simplex virus 2 subunit vaccine elicits a T cell response in mice and is an effective therapeutic vaccine in Guinea pigs. J. Virol. 87, 3930–3942. doi: 10.1128/JVI.02745-12, PMID: 23365421 PMC3624190

[72] Stanfield B. A. Pahar B. Chouljenko V. N. Veazy R. Kousoulas K. G. (2017). Vaccination of rhesus macaques with the live-attenuated HSV-1 vaccine VC2 sti- mulates the proliferation of mucosal T cells and germinal center responses resulting in sustained production of highly neutralizing antibodies. Vaccine 35, 536–543. doi: 10.1016/j.vaccine.2016.12.018, PMID: 28017425

[73] Stanfield B. A. Rider P. Caskey J. Del Piero F. Kousoulas K. G. (2018). Intramuscular vaccination of Guinea pigs with the live-attenuated human herpes simplex vaccine VC2 stimulates a transcriptional profile of vaginal Th17 and regulatory Tr1 responses. Vaccine 36, 2842–2849. doi: 10.1016/j.vaccine.2018.03.075, PMID: 29655629

[74] Stanfield B. A. Stahl J. Chouljenko V. N. Subramanian R. Charles A. S. Saied A. A.. (2014). A single intramuscular vaccination of mice with the HSV-1 VC2 virus with mutations in the glycoprotein K and the membrane protein UL20 confers full protection against lethal intravaginal challenge with virulent HSV-1 and HSV-2 strains. PLoS One 9:e109890. doi: 10.1371/journal.pone.0109890, PMID: 25350288 PMC4211657

[75] Truong N. R. Smith J. B. Sandgren K. J. Cunningham A. L. (2019). Mechanisms of immune control of mucosal HSV infection: A guide to rational vaccine design. Front. Immunol. 10:373. doi: 10.3389/fimmu.2019.00373, PMID: 30894859 PMC6414784

[76] Valerio G. S. Lin C. C. (2019). Ocular manifestations of herpes simplex virus. Curr. Opin. Ophthalmol. 30, 525–531. doi: 10.1097/ICU.0000000000000618, PMID: 31567695 PMC8900730

[77] Van Lint A. L. Kleinert L. Clarke S. R. Stock A. Heath W. R. Carbone F. R. (2005). Latent infection with herpes simplex virus is associated with ongoing CD8+ T-cell stimulation by parenchymal cells within sensory ganglia. J. Virol. 79, 14843–14851. doi: 10.1128/JVI.79.23.14843-14851.2005, PMID: 16282484 PMC1287551

[78] Van Wagoner N. Fife K. Leone P. A. Bernstein D. I. Warren T. Panther L.. (2018). Effects of different doses of GEN-003, a therapeutic vaccine for genital herpes simplex Virus-2, on viral shedding and lesions: results of a randomized placebo-controlled trial. J. Infect. Dis. 218, 1890–1899. doi: 10.1093/infdis/jiy415, PMID: 29982727 PMC7191615

[79] Verjans G. M. Hintzen R. Q. van Dun J. M. Poot A. Milikan J. C. Laman J. D.. (2007). Selective retention of herpes simplex virus-specific T cells in latently infected human trigeminal ganglia. Proc. Natl. Acad. Sci. U. S. A. 104, 3496–3501. doi: 10.1073/pnas.0610847104, PMID: 17360672 PMC1805572

[80] Veselenak R. L. Shlapobersky M. Pyles R. B. Wei Q. Sullivan S. M. Bourne N. (2012). A Vaxfectin(R)-adjuvanted HSV- 2 plasmid DNA vaccine is effective for pro- phylactic and therapeutic use in the Guinea pig model of genital herpes. Vaccine 30, 7046–7051. doi: 10.1016/j.vaccine.2012.09.057, PMID: 23041125 PMC3807597

[81] Villalba M. Hott M. Martin C. Aguila B. Valdivia S. Quezada C.. (2012). Herpes simplex virus type 1 induces simultaneous activation of toll-like receptors 2 and 4 and expression of the endogenous ligand serum amyloid A in astrocytes. Med. Microbiol. Immunol. 201, 371–379. doi: 10.1007/s00430-012-0247-0, PMID: 22622619

[82] Wald A. Koelle D. M. Fife K. Warren T. Leclair K. Chicz R. M.. (2011). Safety and immunogenicity of long HSV-2 peptides complexed with rhHsc70 in HSV-2 seropositive persons. Vaccine 29, 8520–8529. doi: 10.1016/j.vaccine.2011.09.046, PMID: 21945262

[83] Wang K. Dropulic L. Bozekowski J. Pietz H. L. Jegaskanda S. Dowdell K.. (2021). Serum and Cervicovaginal fluid antibody profiling in herpes simplex virus (HSV) Seronegative recipients of the HSV529 vaccine. J. Inf. Dis. 224, 1509–1519. doi: 10.1093/infdis/jiab139 33718970 PMC8599754

[84] Whitbeck J. C. Peng C. Lou H. Xu R. Willis S. H. Ponce de Leon M.. (1997). Glycoprotein D of herpes simplex virus (HSV) binds directly to HVEM, a member of the tumor necrosis factor receptor superfamily and a mediator of HSV entry. J. Virol. 71, 6083–6093. doi: 10.1128/JVI.71.8.6083-6093.1997, PMID: 9223502 PMC191868

[85] Whitley R. J. Green J. W. (2019). “Chapter 350: Herpes Simplex Virus Infections.” in Goldman-Cecil Medicine. ed. L. Goldman (Netherlands: Elsevier), 2189–2192.

[86] Wyles D. L. Patel A. Madinger N. Bessesen M. Krause P. R. Weinberg A. (2005). Development of herpes simplex virus disease in patients who are receiving cidofovir. Clin. Pub. Inf. Dis. Soc. Am. 41, 676–680. doi: 10.1086/432477, PMID: 16080090

[87] Xu X. Zhang Y. Li Q. (2019). Characteristics of herpes simplex virus infection and pathogenesis suggest a strategy for vaccine development. Rev. Med. Virol. 29:e2054. doi: 10.1002/rmv.2054, PMID: 31197909 PMC6771534

[88] Zhang I. Hsiao Z. Liu F. (2021). Development of genome editing approaches against herpes simplex virus infections. Viruses 13:338. doi: 10.3390/v13020338, PMID: 33671590 PMC7926879

[89] Zhang P. Xie L. Balliet J. W. Casimiro D. R. Yao F. (2014). A herpes simplex virus 2 (HSV-2) glycoprotein D-expressing nonreplicating dominant-negative HSV-2 virus vaccine is superior to a gD2 subunit vaccine against HSV-2 genital infection in Guinea pigs. PLoS One 9:e101373. doi: 10.1371/journal.pone.0101373, PMID: 24979708 PMC4076306

[90] Zhu J. Koelle D. M. Cao J. Vazquez J. Huang M. L. Hladik F.. (2007). Virus-specific CD8+ T cells accumulate near sensory nerve endings in genital skin during subclinical HSV-2 reactivation. J. Exp. Med. 204, 595–603. doi: 10.1084/jem.20061792, PMID: 17325200 PMC2137910

[91] Zhu H. Zheng C. (2020). The race between host antiviral innate immunity and the immune evasion strategies of herpes simplex virus 1. Microbiol Mol Biol Rev 84:e00099-20. doi: 10.1128/MMBR.00099-20, PMID: 32998978 PMC7528619

